# Between Gratitude and Responsibility: Reimagining Malaysia’s Healthcare System at a Crossroads

**DOI:** 10.21315/mjms-12-2025-s04

**Published:** 2026-02-28

**Authors:** Mahathar Abd Wahab, Peter Gan Kim Soon, Huan-Keat Chan

**Affiliations:** 1 Director-General of Health Office, Ministry of Health, Putrajaya, Malaysia; 2 Health Transformation Office, Ministry of Health, Putrajaya, Malaysia; 3 Centre for Clinical Trials, National Institutes of Health, Setia Alam, Selangor, Malaysia

**Keywords:** healthcare transformation, delivery of healthcare, healthcare financing, health workforce, public-private partnership

## Abstract

This editorial is written not only from a policymaker perspective, but also as a reflection from citizens who will one day depend on the system we are rebuilding. Malaysia’s public healthcare system, a comprehensive and heavily subsidised national asset built over generations, stands at a critical crossroads. While past investments have yielded equitable access and strong health outcomes, mounting pressures, including a surge in non-communicable diseases, rapid population ageing, and unsustainable medical inflation, now threaten its foundations. Meaningful reform is therefore an act of collective responsibility, not as evidence of past failures. We outline a transformation anchored in three interdependent drivers: (i) sustainable financing through a hybrid model that pools diversified revenues while protecting the poor from catastrophic payments; (ii) integrated service delivery that repositions primary care as the system’s backbone, supported by digital connectivity and strategic public-private partnerships to ensure seamless patient journeys; (iii) a resilient workforce characterized by clearer career pathways, equitable deployment, and genuine investment in well-being. Ultimately, reform succeeds only when it preserves the trust between those who give and those who receive care.

## Introduction

We write this not only as policymakers and healthcare workers, but also as citizen (rakyat) who, like all Malaysians, will one day rely on the healthcare system not in an official capacity, but as patients or caregivers. This dual professional and personal perspective shapes how we view the responsibility of healthcare system stewardship.

Globally, healthcare reform is often discussed in terms of financing models, service delivery structures, or workforce planning (1). Yet at its core, it concerns everyday people at moments of illness, uncertainty, and dependence. Thus, policy choices made by the Malaysian government today will determine whether care remains accessible, timely, and affordable not only for the population at large, but for our own families and communities in the years ahead.

Since independence, Malaysia’s public healthcare system has been built through sustained public investment and a shared societal commitment, and it continues to serve millions with a breadth of services rarely seen in countries at comparable levels of national income. The question before us is therefore not whether the system has value, but whether it is adequately prepared for ongoing epidemiological, demographic, and economic transitions.

In this context, reform is not merely corrective. It is an obligation to anticipate future pressures and to act proactively before constraints become crises. We pursue it not because our healthcare system has failed, but because inaction, in the face of critical and converging challenges, would represent a failure of responsibility to future generations.

## The Global Health Context: Malaysia Is Not Alone

To put things in perspective, healthcare systems worldwide face persistent, multifaceted strain, driven by demographic and disease-pattern shifts, financial constraints, and evolving public expectations. These pressures are not unique to Malaysia; they mirror broader global health trends that test the resilience of healthcare systems across all levels of economic development.

A defining feature of contemporary global health is the rising burden of non-communicable diseases such as cardiovascular disease, cancer, diabetes, and chronic respiratory conditions, which now account for nearly 75% of non-pandemic deaths worldwide (2). Their increasing prevalence, coupled with a global older population now exceeding 700 million (3), places growing demand on health services for chronic care. Meanwhile, climate change is emerging as a major public health threat, projected to contribute an additional 250,000 deaths annually through shifts in vector-borne disease patterns and extreme weather events (4). Population ageing, rising non-communicable diseases, and climate-sensitive health threats are now major concerns in global health planning worldwide.

At the same time, progress toward universal health coverage has stalled since 2015 (5). An estimated 4.6 billion people still lack access to essential health care, while around 1.6 billion are living in or pushed further into poverty due to catastrophic out-of-pocket health expenditures (6). Medical inflation further compounds these pressures, as health spending is expected to continue outpacing global GDP (7), driven by advances in medical technology and rising expectations of care quality.

Even in high-income countries, affordability and access to healthcare remain significant concerns. In the US, where insurance coverage is widespread, around two in five Americans report emotional distress from medical debt, often exceeding that associated with other serious illnesses (8). Despite substantial government investment, the UK National Health Service also continues to face persistent access constraints, with only around 60% of patients treated within the 18-week referral-to-treatment target for elective procedures (9).

While low- and middle-income countries often experience weaker financial risk protection and higher levels of unmet healthcare need, situating Malaysia’s challenges within this broader global context is important. It reinforces that pressure on healthcare systems is now an international norm rather than a national anomaly. It underscores the need for patience, commitment, and public confidence as reforms are pursued.

## Building on Strengths, Facing Key Challenges

Counting our blessings, Malaysia possesses a comprehensive and resilient healthcare system built over generations through the collective commitment of policymakers, healthcare workers, taxpayers, and investors. This national asset is more than a network of hospitals and clinics, combining a functional, dichotomous structure that offers rakyat the choice between public and private care. A highly accessible and equitable public system that serves as our universal health coverage system, ensuring comprehensive care from primary to tertiary levels, with over 3,000 public health clinics and 150 public hospitals nationwide within easy geographic reach of almost all rakyat (10).

The public system is heavily subsidised, enabling millions to access essential services without catastrophic financial expenditures. Amid a steady upward trend, the total health spending reached RM84.2 billion (4.6% of gross domestic product [GDP]) in 2023, with government expenditure accounting for nearly half of it (11). The consistent investments allow the Ministry of Health (MOH) to extend its mandate beyond curative care to include preventive strategies, disease surveillance, and health promotion. Rakyat benefit from pharmaceuticals and diagnostics, dental care, research initiatives, and public health-targeted programmes, for which minimal charges are imposed, as they have been financed through tax-funded and pooled mechanisms, in keeping with global best practices. This combination of breadth, accessibility, affordability, and preventive focus is exceptional internationally. Its success is partly reflected in sustained health outcomes, including a life expectancy exceeding 75 years (12), and maternal and infant mortality rates consistently below the global average (13).

Yet past success does not guarantee future sustainability. Malaysia today faces a rapidly evolving health landscape in line with global trends, with more than half of the population overweight or obese and around one-third living with metabolic syndrome (14). The population is also ageing rapidly, with an estimated 15% of Malaysians expected to be aged 60 years and above by 2030 (15). This demographic shift is associated with a surge in multimorbidity and frailty (16). These conditions drastically increase the complexity of clinical management. Therefore, we can no longer rely on episodic, hospital-centric care to manage patients with multiple chronic illnesses. Such complexity demands a fundamental change in our care models. This complexity demands transformed care models built on seamless integration and continuity across the entire spectrum of settings, from home support to community clinic and hospital.

As medical inflation is also expected to reach 16% in 2026, consistent with global trends acknowledged by the government, escalating healthcare costs and premiums have made access to private care and medical insurance increasingly challenging for many Malaysians (17), thereby increasing demand for public health care services. At the same time, rakyat often perceive healthcare as a right and expect services that are not only timely but also responsive, personalised, and high-quality.

These challenges, unfortunately, amplify the limitations of the Malaysian public health model, which has responded to short-term pressures by becoming increasingly hospital-centric, curative care-focused, reactive, and workforce-intensive. The pressures often manifest in tangible system challenges, including hospital congestion, long waiting times, frequent complaints, an overstrained healthcare workforce, and fragmented yet inefficient services. Importantly, these are structural challenges rather than failures of intent or effort. Recognising them allows reform to be framed as a necessary evolution rather than constant criticism, ensuring that the system can effectively meet both current and future healthcare needs.

## Translating Reform into Action: From Health White Paper to Practical Drivers

**Figure 1:**
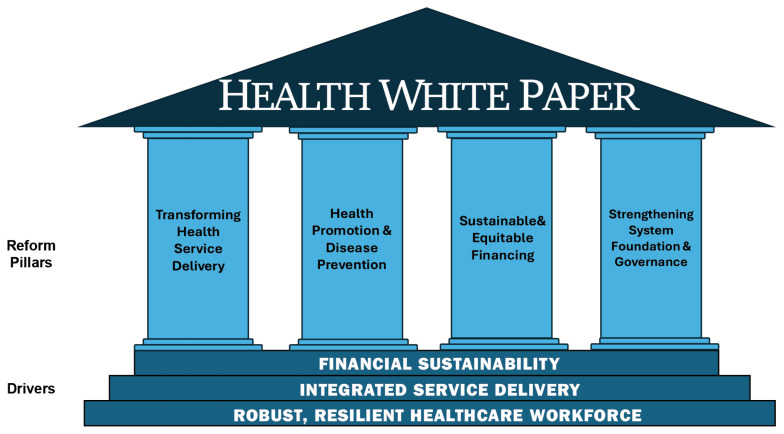
Malaysia Health White Paper

Despite mounting challenges, the MOH has chosen to confront them directly, rather than avoid or deflect them. This commitment is exemplified by the Health White Paper (Figure 1), which outlines a 15-year reform agenda (2023–2038) structured around four pillars that represent a comprehensive vision for a more resilient, accessible, and affordable healthcare system (18). These pillars span: (i) the transformation of service delivery through stronger primary care, hospital optimisation, digital health, and public-private partnership (PPP); (ii) the advancement of health promotion and disease prevention to address non-communicable diseases and other population health risks; (iii) the pursuit of sustainable and equitable health financing to improve affordability and spending efficiency; and (iv) the strengthening of system foundations and governance through institutional reform, workforce development, legislation, and research.

While Health White Paper defines the strategic directions, its implementation ultimately depends on a smaller set of practical drivers that cut across all four pillars, including: (i) financial sustainability; (ii) integrated service delivery; and (iii) a robust, resilient healthcare workforce. These drivers are interdependent yet sequentially actionable, carrying the four pillars forward into day-to-day decision-making and system change. Financial sustainability underpins both service delivery and workforce support; integrated services rely on adequate resources and competent personnel; and a robust, resilient workforce ensures that care is delivered effectively and sustainably. By focusing on these drivers, Malaysia could provide a clear operational roadmap for healthcare reform, bridging the gap between strategic vision and tangible improvements.

## Financial Sustainability: Navigating the Hardest Truth with Caution

Among all the drivers of health system reform, financing is the most difficult to address, the most fundamental and an enabler of integrated service delivery transformation yet. The unavoidable reality is that sustainable health financing cannot be postponed in Malaysia. How healthcare is funded ultimately determines who can access care, how timely and safe that care is, and whether the workforce delivering it can be supported over the long term. Without sound financing, aspirations for equity, quality, and resilience will be unattainable.

Financing reform is complex (19), neither a technical adjustment nor a quick fix. Healthcare financing decisions focus on affordability and accessibility for all Malaysians, ensuring sustainable coverage while maintaining our commitment to free public healthcare. These considerations involve careful planning to balance population needs with fiscal responsibility. Meaningful reform requires societal consensus, political will, careful trade-offs, and a shared understanding of what is sustainable and fair, as informed by global best practices. Moving too quickly or without sufficient engagement may undermine equity and invite pushback.

Therefore, financial reform must be navigated incrementally, guided by evidence on population needs, service use, and outcomes. Encouragingly, local experts are contributing innovative ideas to this discourse (20). For example, a recent concept paper proposes a voluntary National Social Health Scheme with optional, affordable contributions based on individual capacity, designed to complement and strengthen sustained government funding for public healthcare. Such proposals reflect a shared recognition of the need for a more resilient, hybrid financing architecture, one that diversifies revenue, pools risk more effectively and strategically purchases services from both public and private sectors. Some of these ideas could certainly be pursued through rigorous evaluation and dialogue to ensure alignment with existing financing policy.

International experience also offers valuable lessons, both positive and cautionary. Even well-intentioned reforms can falter if changes are perceived as abrupt, poorly explained, or misaligned with public trust. In Greece, rapid austerity-driven cuts to health spending after the financial crisis reduced access, increased unmet medical needs, and worsened population health, illustrating how sudden retrenchment can undermine equity and confidence (21). Similarly, in Chile, attempts to recalibrate private health insurance premiums sparked widespread disagreement, showing how perceived unfairness in financing arrangements can derail reform (22). In contrast, Singapore has navigated healthcare financing to support an ageing population through a combination of mandatory health savings accounts, universal catastrophic insurance, and targeted subsidies, which have been shown to maintain access and financial protection without abrupt shocks to equity or service delivery (23).

These experiences, together with local proposals that emphasise phased pilots, independent governance, and robust public communication, suggest that there is no single model that can be replicated in full, nor any shortcuts that avoid difficult trade-offs. What is clear is that Malaysia’s healthcare refinancing model must adopt a multipronged strategy that reflects the lessons learned from these experiences. At present, the MOH appears to be pursuing a few complementary strategies concurrently at the macro level.

**Figure 2:**
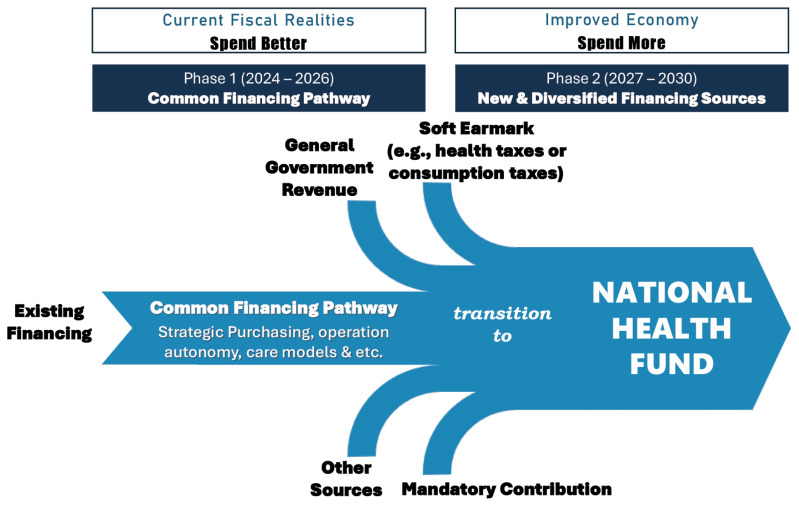
Health financing transformation

One strategy worth highlighting is the reform of public-sector health financing through the National Health Fund (Figure 2), as articulated in the Thirteenth Malaysia Plan (13MP) (24). It aims to pool, diversify, and increase public funding sources while shifting provider payments toward greater accountability for population coverage and health outcomes. The National Health Fund also recognises that financing healthcare for a substantial proportion of the population, including lower-income groups, the unemployed, and older adults without sustainable sources of income, will continue to rely heavily on general government revenues, including general taxation. Therefore, the existing tax-financed anchor for health financing will remain relevant, in keeping with global experiences and best practices, even as sources of healthcare financing are diversified.

Another essential strategy adopts an inter-ministerial approach to address medical inflation, notably by introducing base Medical and Health Insurance/Takaful products with lifetime coverage and more stable premium structures (25). Collectively, these strategies seek to strengthen financial sustainability and advance universal health coverage without imposing excessive fiscal or household burdens. A strengthened financial foundation would also enable the implementation of priority initiatives, including integrated care clusters and targeted benefit packages for vulnerable populations.

Ultimately, financial sustainability is more achievable when reform is paced, transparent, and inclusive, supported by continuous stakeholder engagement. Crucially, necessary changes must be understood by Malaysians as protective rather than punitive, and as investments in their health and well-being rather than withdrawals of support.

## Integrated Service Delivery: Optimising Efficiency

While financial reforms are like skating on thin ice and require whole-of-nation commitments, the optimisation of how care is organised and delivered remains largely within the MOH’s control. Even in the absence of major new funding, reducing fragmentation, optimising existing resources, and ensuring that patients receive the right care, at the right place and at the right time, are practical and impactful approaches. Seamless care across the whole patient journey, from community-based and hospital services to rehabilitation and community reintegration, is critical in this context. Such operational reforms can deliver immediate improvements in access, quality, continuity, and efficiency, while medium to longer-term financial reforms are phased in.

Central to this strategy is the re-orientation of primary healthcare as the backbone of our healthcare system to future-proof our system for an ageing context. Traditional sick-care models are gradually evolving into person-centred, integrated care models within public health clinics, providing comprehensive wellness management, including non-pharmacological interventions and health education. With the growing number of family medicine specialists and multidisciplinary healthcare teams across public health clinics nationwide, this model is increasingly feasible. By decentralising treatment to primary care, patients with manageable conditions can be treated closer to home, reducing unnecessary hospital visits and alleviating the burden on tertiary care.

Concurrently, hospital services are being restructured and optimised to focus on acute and complex care. This strategy ensures that hospital resources and healthcare workers focus on patients who genuinely need tertiary-level inpatient care, while easing congestion, strengthening emergency services, and bolstering day care. Ideas such as one-stop specialist clinic centres can also coordinate multiple treatments in a single visit, reducing repeated hospital attendance or prolonged inpatient admissions. Upgrading secondary hospitals with essential speciality services can strengthen local capacity and reduce unnecessary referrals to overcrowded tertiary centres.

Additionally, care pathways across clinics and hospitals are being redesigned and reengineered to eliminate bottlenecks and reduce fragmentation, streamlining processes from registration and triage to consultation and discharge. Digital tools, including the MyVAS appointment system built on the widely used MySejahtera application, e-Triage innovations, and virtual consultations, are being scaled to shorten waiting times, empower patients with information, and enable remote management of suitable cases. Malaysia is also advancing toward an integrated national electronic medical record system that connects public and private healthcare facilities, adopting a “one patient, one record” approach based on a Master Patient Index. The goal of these digital initiatives is to enable a seamless, efficient, and person-centred care journey, one that respects time and dignity while empowering individuals to be health literate and take ownership of their health.

The private sector, encompassing hospitals, general practitioners (GPs), and support services, is also a critical component of our healthcare system. Strategic PPP will enhance and complement these reforms. By moving beyond a simple public-private dichotomy, targeted collaborations are taking place. Initiatives such as the Hospital Services Outsourcing Programme (HSOP) facilitate resource sharing at reasonable costs (26). The expansion of the Madani Medical Scheme (SPM) involves engaging GPs in patient management (27). These collaborations balance workloads, optimise national healthcare resources across sectors, and improve patient accessibility, reducing the risk of long waits.

All these initiatives underscore a fundamental principle that better utilisation of existing resources can deliver immediate, tangible gains. They are already in progress, and the ultimate measure of success will be the improved experience and feedback from patients across the country.

## Social Determinants of Health: Making the Healthy Choice Easier

**Figure 3:**
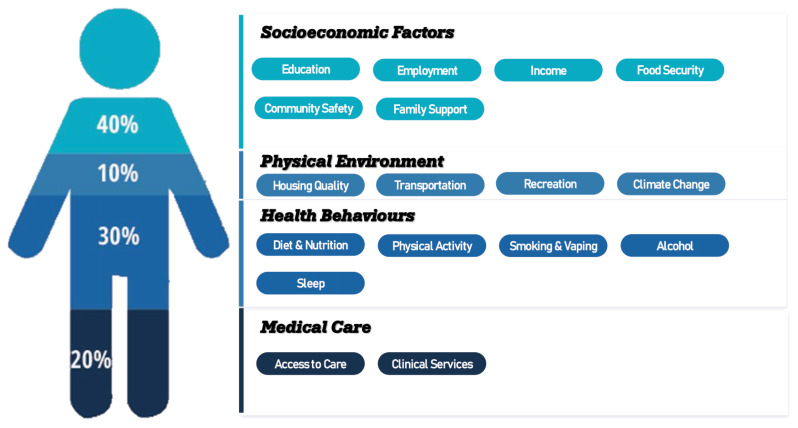
Social determinants of health Adapted from Institute for Clinical Systems Improvement (2014), Going Beyond Clinical Walls: Solving Complex Problems

While improvements in service delivery can enhance healthcare, it is imperative to recognise that most of what shapes health does not occur in clinics or hospitals. It takes place in our homes, schools, workplaces, communities and on roads. Global evidence increasingly shows that medical care accounts for only around 20% of the modifiable contributors to population health outcomes, while the remaining 80% are driven by social, economic, environmental and behavioural conditions that sit essentially outside the formal health sector (28). These social determinants of health (Figure 3) include income security and employment, the quality and accessibility of education, housing and neighbourhood conditions, access to nutritious food, built environment, transport systems, social protection, and the safety of public spaces (29). In practical terms, even a high-performing healthcare system cannot, on its own, fully offset the effects of low wages, insecure work, overcrowded housing, unsafe streets or a food environment saturated with cheap, energy-dense products.

MOH remains responsible for clinical care and public health, but achieving longer, healthier lives for all rakyat requires cross-sector collaboration. Tackling obesity and metabolic risk requires structured collaboration with the Ministry of Education on school food environments, minimum canteen standards and daily opportunities for physical activity. Promoting active living and healthy ageing calls for close partnership with the Ministry of Housing and Local Government and with local authorities so that town plans, public transport, green spaces and safe walking routes support everyday movement. The Health White Paper already recognises this reality by framing health as a whole-of-government and whole-of-society responsibility (18). The central task in the coming decade is to convert that principle into structured, sustained joint action with clear governance and accountability across ministries and sectors.

Preventive care must go beyond clinical interventions to make healthy choices easier for all rakyat, regardless of socioeconomic status. This is supported by measures such as pro-health taxes on sugary-sweetened beverages (30) and other unhealthy products, which raise revenue that can be channelled into the National Health Fund and community-based prevention programmes while reducing risky consumption. The gradual introduction of NutriGrade, a clearer front-of-pack nutrition labelling, is intended to provide consumers with a simple, intuitive guide to sugar (31) and salt content at the point of purchase. The MOH will continue to strengthen clinical and public health services, while championing a “Health in All Policies” approach to embed health into economic, educational, and social policies. Addressing social determinants of health as central reform levers, rather than background factors, is key to reducing non-communicable diseases and shifting from reactive sick care to proactive health care.

## A Robust, Resilient Workforce: The System’s Cornerstone in Need of Delicate Attention

If financing is the health system’s engine and service delivery is its framework, then the workforce is the fuel that drives the system. The dedication of our healthcare workers has carried Malaysia through crises and decades of progress. Nevertheless, acknowledging their indispensable role does not obscure the hard truth that our workforce is under tremendous strain. This is not a reflection of individual failing, but of deep-rooted, structural challenges that threaten the system’s sustainability from within. Addressing these challenges with empathy is, therefore, not merely an operational task; it is a fundamental moral move for reform.

The constant reporting of an overstretched workforce, driven mainly by rising patient demand, increasing case complexity, and uneven distribution that creates critical gaps in certain areas and settings, could not be more accurate. Healthcare professionals, particularly contract doctors and staffs in key schemes, experience employment uncertainty and limited career progression opportunities that impact morale and institutional continuity, which the government and its central agencies are actively addressing through comprehensive workforce reforms. Recognising these challenges, our approach must go beyond mere recruitment, seeking inter-ministerial collaboration to take a holistic view focused on healthcare worker well-being, retention, professional development, and workplace harmony.

The first immediate priority is to achieve a smarter, more equitable and needs-based workforce deployment, with greater emphasis on task-shifting. This involves moving from ad-hoc placements to data-driven workforce planning to align staff distribution with actual community needs and workload. Building a foundation of evidence through robust human resource analytics using appropriate metrics is therefore essential, as it enables the creation of integrated dashboards for real-time monitoring and provides accurate, actionable information. This approach will also support future research into work hours, stress, attrition, and medico-legal risks, paving the way for truly evidence-based human resource management within the MOH.

Second, we aim to pursue clearer career pathways and more dignified remuneration structures. This includes strengthening career pathways and establishing permanent positions for healthcare professionals in critical roles, with structured progression that recognises experiences and expertise. Such an approach will help nurture, retain, and sustain talent within the public healthcare system. In parallel, a careful review of existing incentive frameworks is required to ensure they genuinely reflect the complexity, risk, and demanding nature of healthcare work.

Third, we must enhance the quality and relevance of training, by better aligning education with national needs and promoting collaboration between ministries, public universities, and private training institutions to ensure a competent and accountable workforce. This includes expanding practical, competency-based training delivered under robust supervision, so that skills acquired in training translate effectively into safe, high-quality care in real-world settings.

Fourth, we must make a deliberate effort towards strengthening clinical governance, which is the cornerstone of ensuring accountability for patient safety, delivering high quality care and continuous improvement. Strengthening clinical governance requires embedding a culture of safety, strengthening clinical leadership, and implementing robust, transparent systems for monitoring care. This warrants honesty, transparency and non hierarchical organisational approach to ensure unrestricted feedback and information sharing.

Staff well-being is not a peripheral concern, and staffing pressures within the MOH are tangible and increasingly visible. We are realistic in acknowledging that the public health sector cannot fully match the private sector in terms of income and material incentives. This reality, however, makes it even more imperative to strengthen welfare, job security, and opportunities for professional development of healthcare workers, so that those who choose to remain and serve in the public sector are supported and valued rather than further discouraged.

## Mismatches in Public Health Facilities: Expansion or Back to Basics?

During the transformation journey, the MOH also faces pressure to build more hospitals and health clinics to meet growing healthcare demand. While it is true that some remote rural areas need additional facilities to improve access, indiscriminate expansion can, in most cases, further strain the country’s already limited resources. Our ground visits over the past six months have identified opportunities to optimise resource allocation and further strengthen existing infrastructure across the healthcare network. We are working toward more equitable distribution of medical equipment and systemic upgrading of facilities, from tertiary hospitals to local health clinics. These efforts aim to reduce gaps such as disparities in the age, condition, and availability of medical equipment across different levels of healthcare facilities.

Although upgrading secondary hospitals to include basic specialty services is a feasible way to improve service delivery and access, some specialists have been assigned to these facilities without the necessary support, such as allied health professionals, adequate functional operating theatres, or the appropriate equipment to provide services effectively. Building more facilities without addressing these gaps would likely worsen these mismatches rather than resolve them.

Resource sharing between public, private, and non-governmental organisations is equally crucial in addressing mismatches. While some hospitals operate at full bed capacity and have fully booked operating theatres, others remain underutilised. Rather than just focusing on expansion, priority should be given to establishing strategic partnerships and clustering, including within MOH facilities, with teaching and military hospitals across ministries, and with health institutions under state governments, to maximise the use of existing resources.

Additionally, the current approach to medical equipment maintenance in healthcare facilities warrants re-evaluation. Although the use of concessionaires has been implemented for over two decades and has yielded measurable efficiency gains (32), their scope of responsibility and technical expertise, particularly in relation to increasingly complex and technologically advanced equipment, require reassessment to determine whether they can adequately keep pace with evolving demands. In this context, greater involvement of the manufacturers and authorised dealers may serve as a complementary strategy to enhance maintenance efficiency, given their superior technical knowledge and access to proprietary tools. Furthermore, procurement models, including conventional outright purchasing, lease-to-use arrangements, and potential hybrid approaches, should be systematically evaluated to identify the best mechanisms that best support sustainable and cost-effective service delivery.

## Data-Driven Quality Oversight: Completing the Puzzle

It is essential to confront challenges directly, and one key approach is to be honest and transparent with ourselves by establishing key performance indicators (KPIs) that genuinely reflect service quality and progress of healthcare transformation at a macro level. Rather than taking a cautious approach and setting easily achievable targets, it is timely to face the real issues. One key lesson from other countries suggests the importance of defining or revising KPIs around critical wait times.

For instance, there is currently no standardised measure for referral-to-treatment time in Malaysia. Internal data indicate that most procedural wait times across a range of disciplines, including surgery, obstetrics and gynaecology, radiotherapy and oncology, respiratory care, and psychiatry, generally meet the targets we have set. However, there are notable exceptions: only 72.1% of patients with suspected gastrointestinal malignancy received endoscopy within four weeks, compared with a target of 80%. Achieving these targets does not necessarily suggest that our KPIs are comprehensive or that the targets themselves are appropriate. Wait time standards may also vary across settings, as a recent internal survey shows that patients in high-volume hospitals may wait up to six months for elective procedures.

Beyond procedural wait times, other critical indicators, including ambulance response times, bed availability, and outpatient waiting periods, are frequently sources of patient dissatisfaction and require reassessment. In the coming months, the focus should be on revisiting and refining our existing KPIs, not only for wait times but also for a broader set of indicators that accurately reflect healthcare system performance and patient experience. In this context, we can leverage the digital platforms already in place to gather and analyse this data.

## Conclusion

Healthcare reform is ultimately not about systems or structures, but about people, including those who seek care today and those who will depend on it in the years to come. Therefore, the responsibility for reform must be shared by all Malaysians and cannot rest solely with the government. It requires sustained leadership from the healthcare institutions, active partnership with healthcare professionals across disciplines and sectors, and the engagement of the public we serve. The guiding principle should always be collaboration and returning to basics to consolidate what we already have, with digitalisation and sustainable financing serving as key catalysts.

Progress will neither be linear nor immediate. Meaningful reform is a long journey, one that demands openness and the humility to acknowledge limitations while remaining committed to improvement. The MOH welcomes recommendations and constructive criticism, not as a challenge to what has been built, but as a means to renew our healthcare system responsibly and collectively. Equally, the MOH seeks the trust of all stakeholders, recognising that trust is the very “thrust” of healthcare reform, a commitment we uphold through consistent honesty, transparency and accountability.
